# Antileukemic Activity of Sulforaphane in Primary Blasts from Patients Affected by Myelo- and Lympho-Proliferative Disorders and in Hypoxic Conditions

**DOI:** 10.1371/journal.pone.0101991

**Published:** 2014-07-14

**Authors:** Carmela Fimognari, Eleonora Turrini, Piero Sestili, Cinzia Calcabrini, Giovanni Carulli, Giulia Fontanelli, Martina Rousseau, Giorgio Cantelli-Forti, Patrizia Hrelia

**Affiliations:** 1 Department for Life Quality Studies, Alma Mater Studiorum-University of Bologna, Rimini, Italy; 2 Department of Biomolecular Sciences, University of Urbino "Carlo Bo", Urbino, Italy; 3 Division of Hematology, Department of Clinical and Experimental Medicine, University of Pisa, Pisa, Italy; 4 Department of Pharmacy and BioTechnology, Alma Mater Studiorum-University of Bologna, Bologna, Italy; Università degli Studi di Firenze, Italy

## Abstract

Sulforaphane is a dietary isothiocyanate found in cruciferous vegetables showing antileukemic activity. With the purpose of extending the potential clinical impact of sulforaphane in the oncological field, we investigated the antileukemic effect of sulforaphane on blasts from patients affected by different types of leukemia and, taking into account the intrinsically hypoxic nature of bone marrow, on a leukemia cell line (REH) maintained in hypoxic conditions. In particular, we tested sulforaphane on patients with chronic lymphocytic leukemia, acute myeloid leukemia, T-cell acute lymphoblastic leukemia, B-cell acute lymphoblastic leukemia, and blastic NK cell leukemia. Sulforaphane caused a dose-dependent induction of apoptosis in blasts from patients diagnosed with acute lymphoblastic or myeloid leukemia. Moreover, it was able to cause apoptosis and to inhibit proliferation in hypoxic conditions on REH cells. As to its cytotoxic mechanism, we found that sulforaphane creates an oxidative cellular environment that induces DNA damage and Bax and p53 gene activation, which in turn helps trigger apoptosis. On the whole, our results raise hopes that sulforaphane might set the stage for a novel therapeutic principle complementing our growing armature against malignancies and advocate the exploration of sulforaphane in a broader population of leukemic patients.

## Introduction

Sulforaphane (SR) is a dietary isothiocyanate found in cruciferous vegetables able to provide protection against multistep carcinogenesis [1]. Epidemiological studies evidenced an inverse correlation between the consumption of a diet rich in cruciferous vegetables (i.e., broccoli and cabbage) and the incidence of breast, lung, prostate, colon, and bladder cancer [2]–[7], largely attributed to the activity of isothiocyanates derived from the metabolism of glucosinolates that accumulate in cruciferous vegetables [8]. SR is a highly reactive and hydrophobic compound that can alter many cellular processes. Inhibition of cell proliferation, increased apoptosis, anti-inflammatory and antioxidant activities, induction of phase-II detoxification enzymes, inhibition of cyclooxygenase 2, and various other mechanisms have been proposed to explain the anticancer effects of SR [9].

SR induces apoptosis in several cancer cell lines, such as T-cell leukemia, breast, colon, and prostate cancer, by targeting different molecules, such as caspases, PARP, p21, p53 and Bax [10]–[14]. SR also blocks cell cycle through the modulation of G1/S and G2/M phases and alters the levels of cyclin A, cyclin B1, cyclin D1, p21cip1/waf1, and KLF4 [12], [15]–[19]. The antileukemic effect of SR was demonstrated in many different cell lines and, recently, also in blasts from pediatric patients with acute lymphoblastic leukemia (ALL) [20].

Leukemias are malignant neoplasms involving cells originally derived from hematopoietic precursor cells that include many diverse and biologically distinct subgroups. All leukemias start in the bone marrow, that is diffusely replaced by abnormally proliferating neoplastic cells. The neoplastic cells may spill out of the bone marrow and reach the blood, where they may be present in large numbers, resulting in the clinical presentations of the disease. Generally, the leukaemias can be divided into acute myeloid leukemia (AML) and related disorders, B-lymphoblastic leukemias, T-lymphoblastic leukemias, leukemias of ambiguous lineage [21].

Although leukemia is the most common malignancy among children and adolescents, the majority of cases of leukemia occur in older people [22].

The treatment of leukemia still largely revolves around chemotherapy to induce a complete remission and to consolidate this with further cycles of chemotherapy. For example, the standard therapy of AML is based on the association of an anthracycline and ARA-C and the efficacy of such therapy still is unsatisfactory, since the rate of complete remission ranges 30–60%, depending on age, and the survival rate has not changed significantly in years [23].

Several new therapeutic approaches are under investigation, alone or in combination with conventional chemotherapy. Despite the development of multiple new agents, the development of chemoresistance frequently hampers the successful treatment of acute and chronic leukemias either at the initial presentation or (more frequently) following primary or subsequent relapses [24], and relapse continues to be the most common cause of death [25]. Those therapeutic issues can also be imputable to tumor microenvironment, which is characterized not only by marked gradients in drug concentration, but also by regions of hypoxia, which can influence tumor cell sensitivity to drug treatment. Taking into account the intrinsically hypoxic nature of bone marrow, hypoxia is also an important environmental factor in leukemia [26].

Thus, leukemias remain a formidable therapeutic challenge that requires the identification and the development of novel agents for the treatment of this disease.

Based on these considerations and with the aim to extend the potential clinical impact of SR in the oncological field, we investigated whether purified SR is effective against hematological malignancies. To this end, we explored the antileukemic effect of SR on blasts from patients affected by different types of leukemia. In particular, we tested SR on patients with chronic lymphocytic leukemia (CLL), AML, T-cell ALL (T-ALL), B-cell ALL (B-ALL), and blastic NK cell leukemia (BNKAL). Since SR activity was marked on samples from acute leukemias, we next analyzed the proapoptotic activity of SR in two acute leukemia cell lines. To better understand whether the level of oxygen is relevant for the therapeutic efficacy of SR, we treated a leukemia cell line with SR in hypoxic conditions and analyzed the formation of ROS and the induction of apoptosis.

## Materials and Methods

### Chemicals

Ethidium bromide, NaOH, 2′,7′-dichlorofluorescein-diacetate (DCFH-DA), chloroform, phenol, NaH_2_PO_4_ were purchased from Sigma (USA). Synthetic DL- SR was dissolved in dimethyl sulfoxide (DMSO) to generate a 40 mM stock concentration and stored at −20°C. Cells were treated with different concentrations of SR (0.0–30.0 µM), selected on the basis of previous studies performed on leukemia cells [15].

### Leukemic cell lines

REH (acute lymphocytic leukemia) and HL-60 (acute promyelocytic leukemia) cell lines were bought from ATCC-LGC, grown in suspension and propagated in RPMI 1640 supplemented with 10% (REH) or 20% (HL-60) heat-inactivated bovine serum, 1% antibiotics (all obtained from Sigma). To maintain exponential growth, the cultures were divided every third day by dilution to a concentration of 1×10^5^ cells/mL. Cells were treated with different concentrations of SR for different times at 37°C in both normoxic (20% O_2_) and hypoxic conditions.

### Hypoxic conditions

Leukemic cells were cultivated in an INVIVO_2_ 200 hypoxia workstation (Ruskinn Technology LTD, England) at <1% O_2_. Treatments and all cell manipulations were performed in the workstation, thus ensuring a full maintenance of hypoxia for the entire duration of the experiments.

### Ethics statement

The described study was approved (Comitato Etico e Sperimentazione del Farmaco dell'Azienda Ospedaliero-Universitaria Pisana) and written informed consent was obtained from the patients. All clinical investigation was conducted according to the principles expressed in the Declaration of Helsinki.

### Hematologic patients

Patients include cases of CLL (n = 2), AML (n = 8), B-cell ALL (n = 1), T-cell ALL (n = 2), BNKAL (n = 1). The general characteristics of patients are shown in Table 1. Diagnosis of leukemia was established according to the 2008 WHO classification [21] and by the combination of morphological, immunological, cytogenetic and molecular methods, which were applied to peripheral blood samples. The immunological assays were made by fluorochrome-conjugated monoclonal antibodies and analysis by a three-laser (488, 633, 405 nm)-equipped flow cytometer (FacsCanto II, Becton Dickinson, USA). A six-color method was applied; therefore the following fluorochrome combination was used: fluorescein isothiocyanate, phycoerythrin, peridinin chlorophyll protein complex, phycoerythrin-cyanine 7, allophycocyanin, allophycocyanin-cyanine 7. Diagnosis of acute leukemia was established by means of a wide panel of monoclonal antibodies, which included: CD45, CD13, CD33, CD34, CD117, HLA-DR, CD4, CD14, CD64, CD38, MPO, CD11b, CD16, CD15, CD56, CD7, CD19, CD3, CD2, CD5, CD4, CD8, CD10, CD20, CD58, TdT (from Becton Dickinson) and rabbit polyclonal F(ab′)2 antibodies directed to cyIgM and surface immunoglobulin K and lambda chains (from Dako, USA). CLL samples were subjected to an antibody panel which included CD45, CD3, CD4, CD8, CD16-56, CD5, CD19, CD20, CD23, CD22, CD79b, CD200, CD38, CD25, CD11c, surface immunoglobulin K and lambda chains. Patients were studied at the time of diagnosis; one patient was studied during his first relapse and two patients in a phase of resistant disease.

**Table 1:** Clinical features of patients.

Patients	Age	Sex	Diagnosis	Timing	Previous therapy
1	67	F	CLL	Stable disease	None
2	76	F	CLL	Stable disease	None
3	38	M	AML	Studied at diagnosis	None
4	39	M	AML	Non-responder	3+7^a^
5	31	F	AML	Non-responder	3+7
6	19	F	AML	Studied at diagnosis	None
7	30	F	AML	Studied at diagnosis	None
8	60	F	AML	Relapse	3+7, allogeneic HSCT^b^
9	47	F	AML	Studied at diagnosis	None
10	24	M	T-ALL	Studied at diagnosis	None
11	24	F	T-ALL	Studied at diagnosis	None
12	48	M	B-ALL	Studied at diagnosis	None
13	55	F	AML	Studied at diagnosis	None
14	31	F	BNKAL	Studied at diagnosis	None

a a combination drug protocol used as induction chemotherapy and consisting of three days of anthracyclines and seven days of cytarabine;

b allogeneic HSCT: transplantation of allogeneic hematopoietic stem cells.

### Preparation of leukemic cells

Peripheral blood samples of patients were collected in tubes containing preservative-free heparin. Leukemic cells were obtained by Ficoll-Histopaque density gradient centrifugation. 3 mL of Histopaque 1.077 g mL^−1^ were placed into a 10 mL plastic centrifuge tube, overlaid with 3 mL anticoagulated blood diluted 1∶1 with phosphate-buffered saline (PBS), and centrifuged at 400×g for 30 min at room temperature. Interphase mononuclear cells banded at the interface between the plasma and the Histopaque were recovered, washed twice with PBS and then resuspended in RPMI 1640 medium (Sigma) containing 15% heat-inactivated bovine serum. The samples always contained >95% blasts.

### Flow cytometry

All flow cytometric analyses were performed by using the easyCyte 5HT flow cytometer (Millipore Guava Technologies, USA).

### Detection of apoptosis

After 24, 48 or 72 h of treatment with different concentrations of SR, aliquots of 2.0×10^4^ cells were stained with 100 µL of Guava Nexin Reagent containing ANNEXIN-V-phycoerythrin and 7-amino-actinomycin D. During apoptosis, the cells react to annexin V once chromatin condenses but before the plasma membrane loses its ability to exclude 7-amino-actinomycin D. Hence, by staining cells with a combination of phycoerythrin annexin V and 7-amino-actinomycin D it is possible to detect non apoptotic live cells, early apoptotic cells and late apoptotic or necrotic cells. Cells were incubated for 20 min at room temperature in the dark and then analyzed by flow cytometry.

### Fast halo assay (FHA)

The assay has been carried out as previously described [27], [28]. Briefly, after the treatments, the cells were resuspended at 4.0×10^4^/mL in ice-cold PBS containing 5 mM EDTA: 25 µL of this cell suspension was diluted with an equal volume of 2% low melting agarose in PBS and immediately sandwiched between an agarose-coated slide and a coverslip. After complete gelling on ice, the coverslips were removed and the slides were immersed in NaOH 300 mM for 15 min at room temperature. Ethidium bromide (10 µg/mL) was directly added to NaOH during the last 5 min of incubation. The slides were then washed and destained for 5 min in distilled water. The ethidium bromide-labelled DNA was visualized using a Leica DMLB/DFC300F fluorescence microscope (Leica Microsystems, Germany) equipped with an Olympus Colorview IIIU CCD camera (Olympus Italia Srl, Italy) and the resulting images were digitally recorded on a PC and processed with an image analysis software (Scion Image, Scion Corporation, USA). The amount of fragmented DNA diffusing out of the nuclear cage, i.e. the extent of strand scission, was quantified by calculating the nuclear diffusion factor (NSF), which represents the ratio between the total area of the halo and nucleus and that of the nucleus. To allow the detection and recognition of DNA double strand breaks (DSBs) FHA was carried out at non-denaturing pH conditions as follows: the slides were submersed in a lysis solution (0.15 M NaOH, 0.1 M NaH_2_PO_4_, 1 mM EDTA, Triton ×100 1% v/v, pH 10.1) for 10 min, incubated for further 15 min in PBS (pH 7.4) containing 0.1 mg/mL RNase (bovine pancreas Type 1A); ethidium bromide was directly added to this solution during the last 5 min of incubation. The slides were then washed and destained for 5 min in distilled water and analyzed as described above.

### ROS detection

DCFH-DA was used for ROS detection. DCFH-DA is cleaved intracellularly by nonspecific esterases to form 2′,7′-dichlorodihydrofluorescein (DCFH), which is further oxidized by ROS to form the highly fluorescent compound 2′,7′-dichlorodihydrofluorescein (DCF). Briefly, 1×10^6^ cells were pre-treated with 15 or 30 µM SR for 30, 60, 180 or 360 min. Then, samples were washed and 1×10^6^ cells were stained with 10 µM DCHF-DA. After 20 min of incubation at 37°C, fluorescence intensity was monitored by flow cytometry. Data were expressed as percentage of the control (untreated cells).

### Analysis of apoptotic proteins

After treatment with SR, 1×10^6^ cells were fixed and permeabilized by 2% of paraformaldehyde in PBS 1× and 90% of cold methanol. They were then incubated with Bax (1∶100, Santa Cruz Biotechnology, Santa Cruz, CA, USA), Bcl-2 (1∶100, Santa Cruz Biotechnology), p53 (1∶100, Invitrogen), or isotype-matched negative control (1∶100, e-Bioscience, San Diego, CA, USA) antibodies. The cells were washed and incubated with fluorescein isothiocyanate-labeled secondary antibody (1∶100, Sigma). The cells were then analyzed to quantify fluorescein isothiocyanate binding by flow cytometry. Mean fluorescence intensity values were calculated. Non-specific binding was excluded by gating around those cells which were labeled by the fluorescein isothiocyanate-conjugate isotype control.

### Analysis of cell cycle

Cell were treated with different concentrations of SR for 8, 24 and 48 h, and then fixed with ice-cold ethanol. Samples were then stained with 200 µL of Guava Cell Cycle Reagent, containing propidium iodide that allows evaluating cellular DNA content. During the S phase of cell cycle, cells duplicate their content of DNA that will be double in the G_2_/M phase compared to the G_0_/G_1_ phase. Cells were incubated at room temperature for 30 min in the dark, and analyzed via flow cytometry.

### mRNA expression

After 6, 24 or 48 h of treatment in normoxic or in hypoxic conditions, total RNA was isolated using miRVana™ miRNA Isolation kit (Life Technologies, CA, USA), according to manufacturer's instructions. Briefly, cells were treated with lysis buffer, then subjected to acid - phenol: chloroform extraction. Ethanol was added to samples and they were passed through a filter cartridge containing a glass-fiber filter which immobilizes the RNA. The filter was washed and RNA was eluted with a low ionic strength solution. Total collected RNA was used for reversed transcription by High Capacity cDNA Reverse Transcription kit (Life Technologies). Briefly, 200 ng total RNA were added to 10 µL reaction kit mixture with RNase inhibitor according to manufacturer's instructions as well as the thermal cycler conditions. The obtained cDNA was stored at −20°C. Quantification of Bax, Bcl-2, TP53 and 18 S, as endogenous control, was performed in triplicate by real-time PCR (ABI Prism 7900HT, Life Technologies), using Universal Master Mix and TaqMan assays Hs00180269_m1 (Bax), Hs00608023_m1 (Bcl-2), Hs01034249_m1 (p53) and Hs99999901_s1 (18S) (Life Technologies). Each measurement was conducted in triplicate.

### Statistical analysis

All results are expressed as the mean ± SEM of at least four different experiments. Differences in mRNA expression are reported as value ± SEM and represent the relative expression calculated through the 2^-ΔΔC^t method [29]. One way ANOVA, followed by Dunnett or Bonferroni as post test was used to evaluate differences between treatments. GraphPad InStat version 5.0 (GraphPad Prism, San Diego, CA, USA) was used for all statistical analyses. P<0.05 was considered significant.

## Results

**Figure 1:**
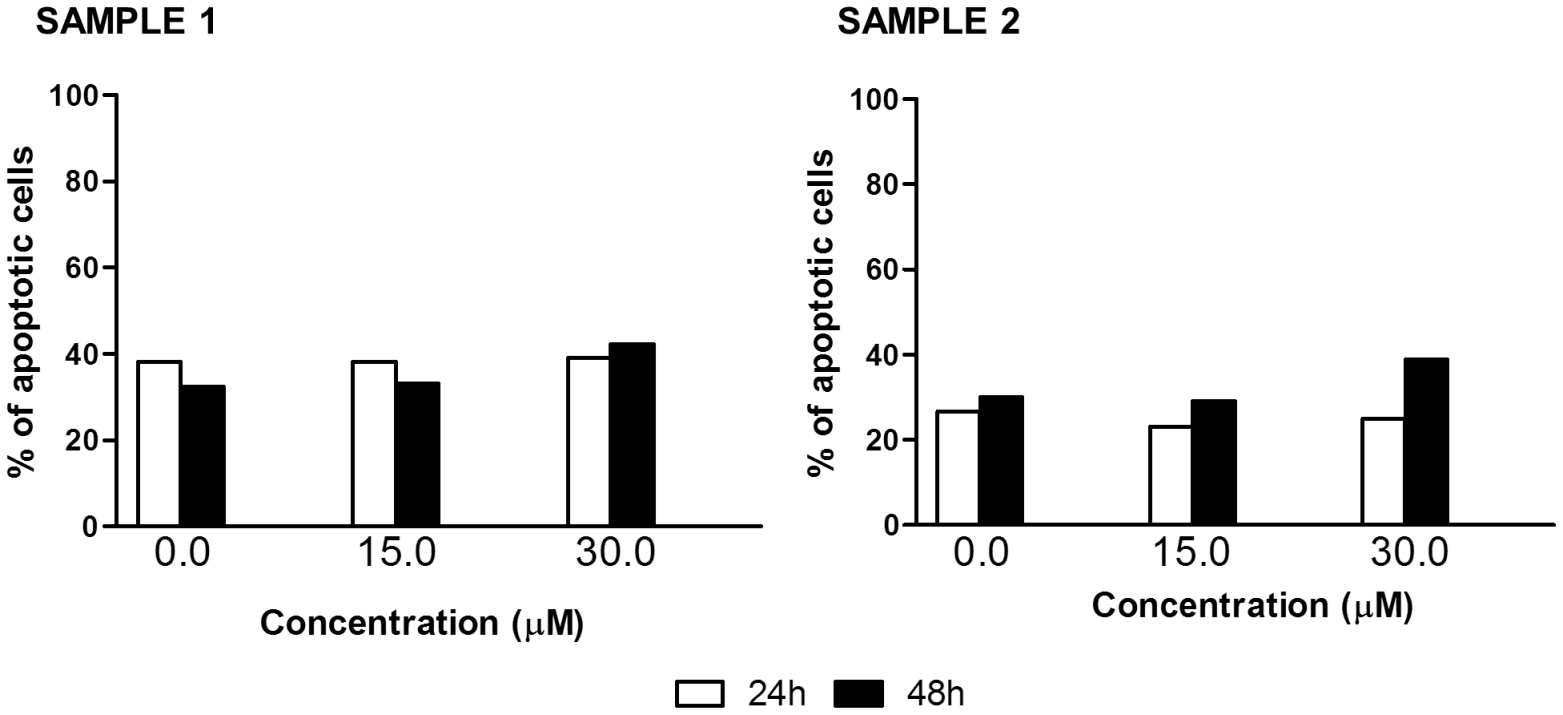
Fraction of apoptotic cells induced by SR on mononuclear cells isolated from CLL patients. Cells were treated with 0 to 30 µM SR for 24 or 48 h.

Firstly, we examined the effects of different concentrations of SR on primary blasts collected from leukemic patients. Three samples (patients n. 6, 7 and 8) were excluded because they had a % of viable cells lower than 10% when untreated. SR seems not to have any activity on samples from patients with CLL. Even high concentrations (30 µM) and long times (48 h) of treatment only slightly increased the % of apoptotic cells compared to the control (42.3% vs. 32.4%, sample n. 1) (Fig. 1).

**Figure 2:**
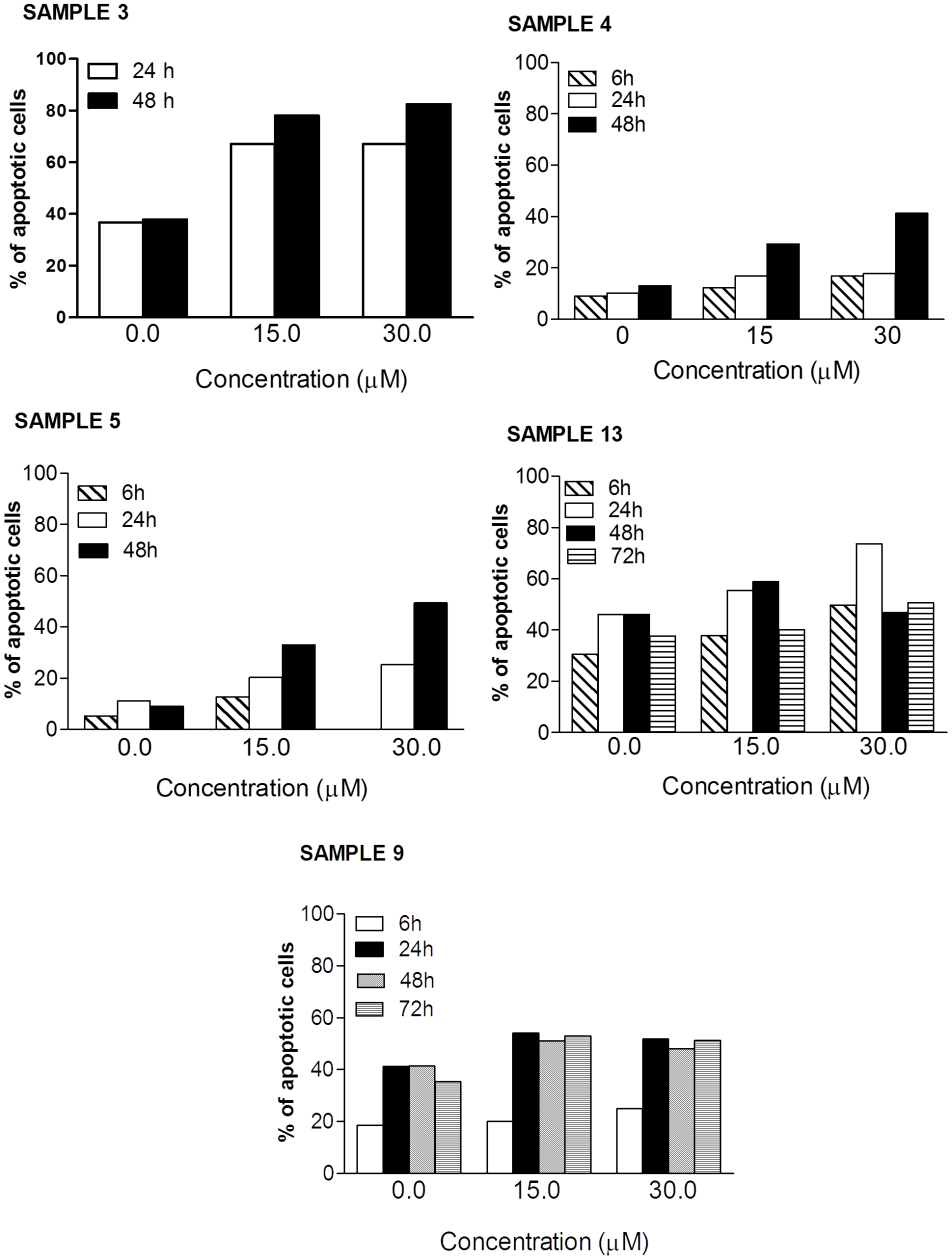
Fraction of apoptotic cells induced by SR on mononuclear cells isolated from AML patients. Cells were treated with 0 to 30 µM SR for 6, 24, 48 or 72 h.

The pro-apoptotic effect of SR was recorded, however, in samples from patients with AML, where the % of apoptotic cells induced following treatment reached 49% (sample n. 5) (Fig. 2). Both samples 3 (about 45% apoptosis increase vs. control at 30 µM after 48 h incubation) and 5 (about 40% apoptosis increase vs. control at 30 µM after 48 h incubation) were highly sensitive to SR treatment (Fig. 2). In contrast, samples 4 (about 28% apoptosis increase vs. control at 30 µM after 48 h incubation), 13 (about 28% apoptosis increase vs. control at 30 µM after 24 h incubation), and 9 (about 13% apoptosis increase vs. control at 15 and 30 µM after 24 h incubation) were less sensitive (Fig. 2). Of note, the effect of SR was marked also on samples from non-responder patients. In the sample n. 5, for example, the fraction of apoptotic cells recorded after 48 h of treatment with 30 µM SR is more than 5 times higher than that recorded in untreated cultures (Fig. 2).

**Figure 3:**
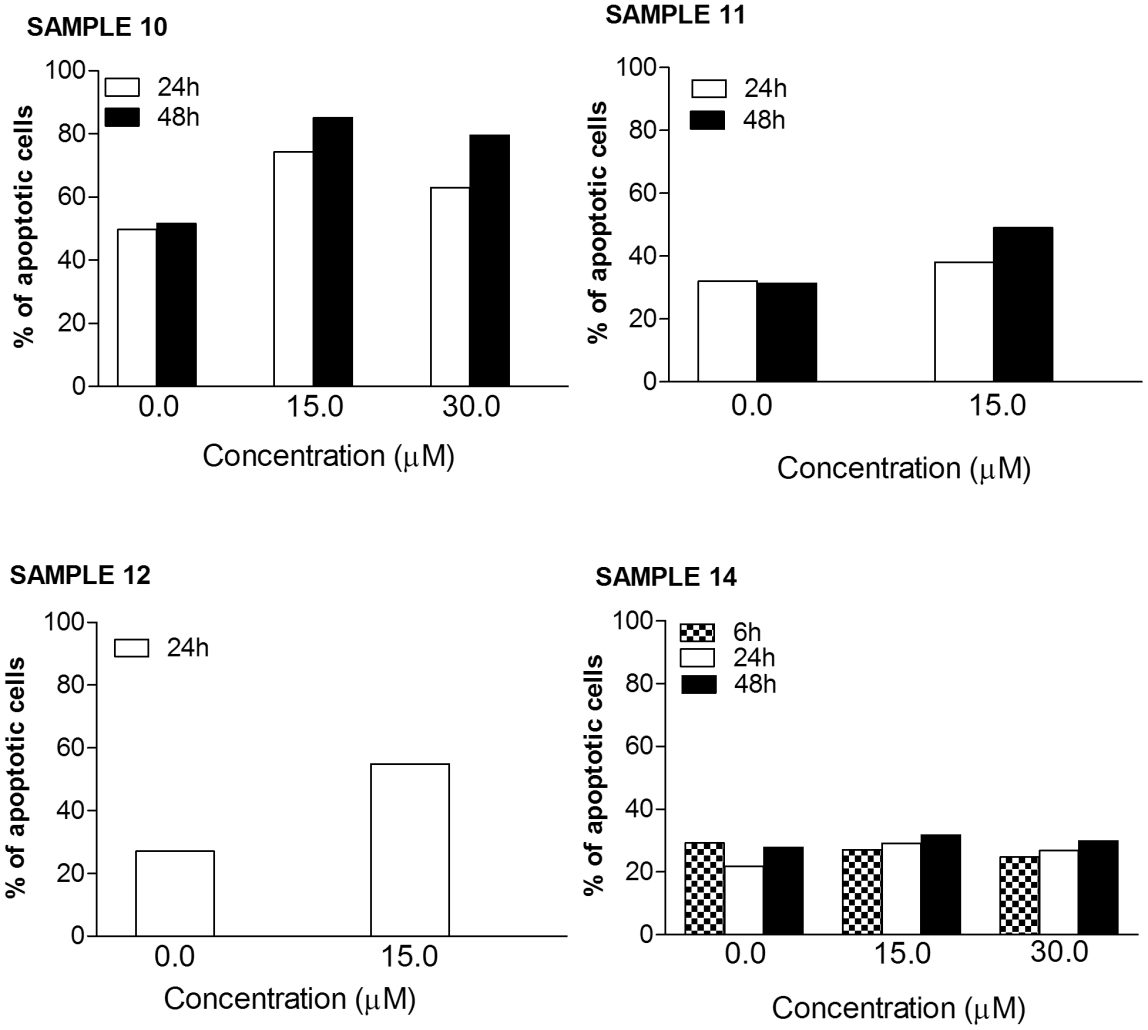
Fraction of apoptotic cells induced by SR on mononuclear cells isolated from ALL and BNKAL patients. Cells were treated with 0 to 30 µM SR for 6, 24 or 48 h.

The activity of the isothiocyanate on samples from patients with ALL did not differ between T- or B- cell leukemia. The most marked effect was definitely observed on the sample from patient suffering from T-ALL, where the % of apoptotic cells reached 55% (vs. 27% in the control cultures) (sample n. 10) (Fig. 3). The effect on the other sample of T-ALL was milder (sample n. 11) (about 20% apoptosis increase vs. control at 15 µM after 48 h incubation) (Fig. 3). A 28% increase in the apoptotic effect was reached in the B-ALL sample at 15 µM after 24 h incubation (sample n. 12) (Fig. 3).

SR has not been shown to have any activity on the sample from BNKAL (sample n. 14) (Fig. 3).

**Figure 4:**
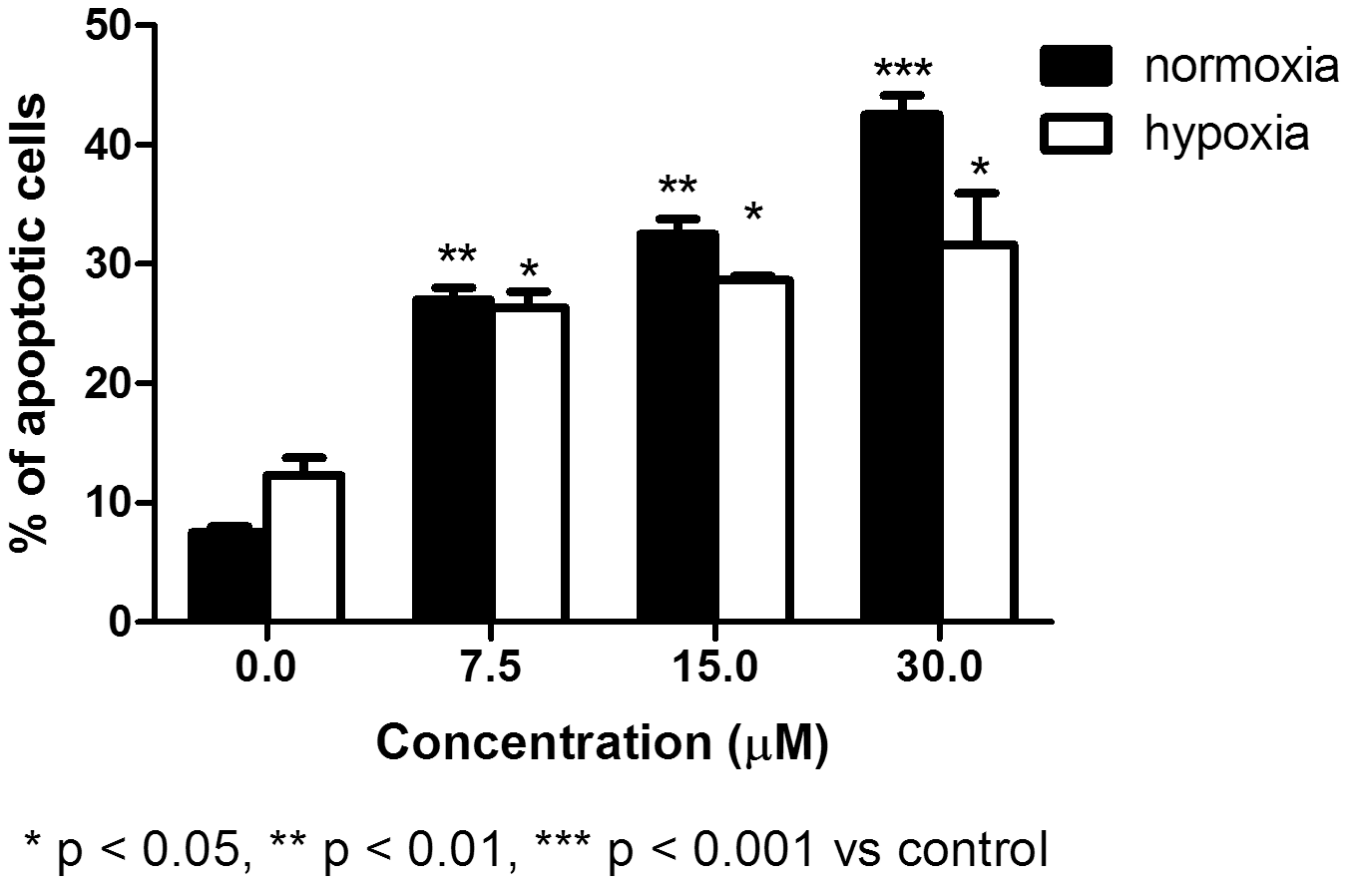
Induction of apoptosis by SR in normoxic and hypoxic conditions on REH cells. Cells were treated with 0 to 30 µM SR for 24 h. The % of apoptotic cells recorded in the untreated cultures was subtracted from that observed in cultures treated with SR. Data are presented as mean ± SEM of at least four different experiments.

We next analyzed the proapoptotic activity of SR and initially determined the induction of apoptosis after treating two acute leukemia cell lines with different doses of SR for 24 h. In REH cells, we observed a clear increase in the fraction of apoptotic cells under all conditions (Fig. 4). Similar results were observed in HL-60 cells, where we recorded a two-fold increase with respect to controls after 48 h of treatment.

**Figure 5:**
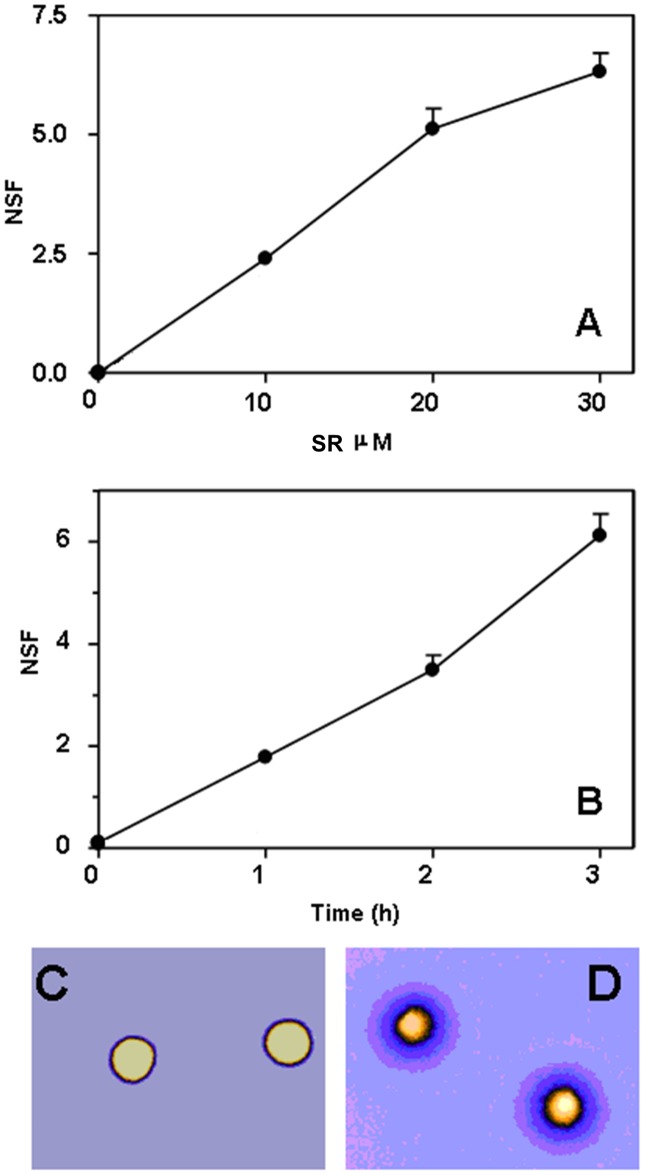
SR induces DNA damage in REH cells. Cells were treated with increasing concentrations of, or for increasing time intervals with, SR and immediately assayed for DNA breakage with FHA. A) DNA single strand breakage induced by 3 h SR treatment in REH cells. B) DNA single strand breakage induced by 30 µM SR in REH cells as a function of incubation time. Data are presented as mean ± SEM of three different experiments. C) and D) Representative, digitally pseudocolored (ICA look up table of the Image J software) micrographs of FHA-processed control (C) or 30 µM SR-treated (D) REH cells are also shown: note the wide halos in D as compared to C. Also shown (panel A, inset) the extent of DNA double strand breakage caused by SR (30 µM for 3 h) or etoposide (1 µg/mL for 3 h) in REH cells. Data are expressed as NSF (nuclear diffusion factor), which represents the ratio between the total area of the halo and nucleus and that of the nucleus.

Genomic DNA breaks represent an important trigger of apoptosis. Thus, we investigated the effect of SR on REH cells. REH cells were exposed to SR (10, 20 and 30 µM) for 3 h, and analyzed immediately for direct DNA strand scission using the sensitive FHA. With this technique, DNA fragments resulting from the cleavage of DNA itself diffuse out of the nuclear cage as an inverse function of their size, thus producing a concentric halo whose radius reflects the extent of DNA damage. Halo formation was monitored at the single cell level with fluorescence microscopy, as seen in the representative micrographs shown in Fig. 5C–D. The results obtained indicate that SR promotes the formation of DNA breaks in a dose-dependent fashion (Fig. 5A). Formation of DNA breaks as a function of treatment time with 30 µM SR is linear over time (Fig. 5B).

We then explored the nature of SR-induced DNA lesions by analyzing the nuclear DNA of treated cells at lower -non denaturing conditions- lysis pH values, i.e. 9.3. Indeed, while operating at pH 13.00 does not allow to distinguish DNA single strand breaks (SSBs) from DSBs, at pH 9.3 nuclear DNA is not hydrolyzed into the two chains and only the DNA fragments resulting from double strand cleavage can be detected. Under these latter conditions (i.e. pH 9.3), no difference in terms of DNA fragmentation could be observed between treated (30 µM SFN for 3 h) and untreated REH cells (Fig. 5A, inset), thus suggesting that the lesions caused by SFN observed at pH 13.00 are frank SSBs. On the contrary, treatment of REH cells with etoposide (1 µg/ml for 3 h), included as a representative inducer of DNA DSBs, resulted in extensive DNA double strand breakage (Fig. 5A, inset).

**Figure 6:**
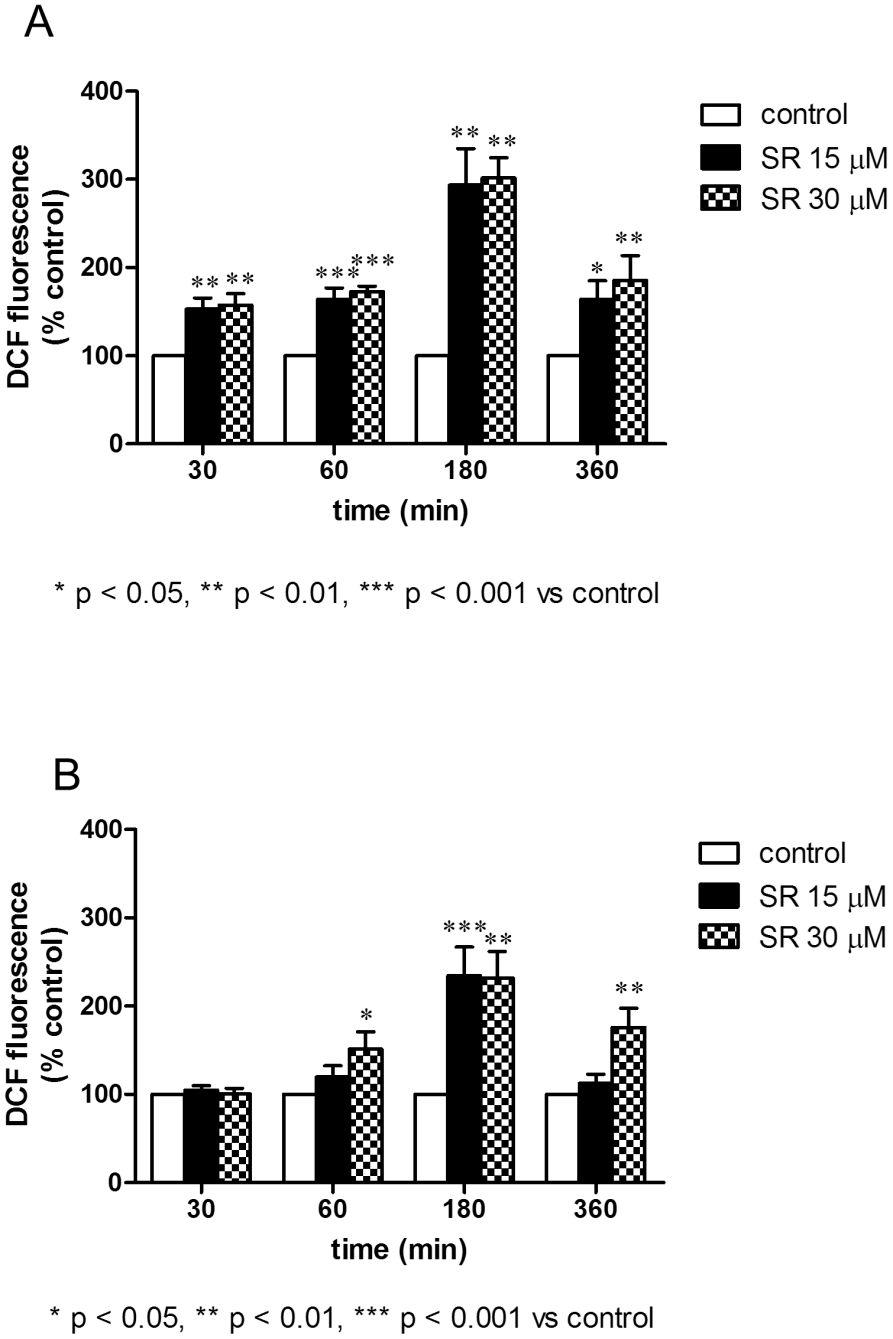
Effect of SR on ROS levels determined in normoxic (A) and hypoxic (B) conditions on REH cells. Cells were treated for 30–360 min with SR to analyze the oxidation state of the cell by using DCFH-DA as fluorogenic probe. Results are expressed as DCF (2′,7′-dichlorodihydrofluorescein) fluorescence (% of control) and are means ± SEM of four independent experiments.

As to the mechanism responsible for the induction of DNA damage in SR-treated cells, we previously reported that SR promotes the mitochondrial formation of ROS [30], which are the ultimate species mediating DNA cleavage. We then investigated the formation of ROS in REH cells. After 3 h of treatment with SR 30 µM, we recorded a significant induction of ROS. Results depicted in Fig. 6 indicate that SR caused the conversion of DCFH into its fluorescent by-product, a process which reflects the formation of ROS. Treatment of cells with SR greatly increased the formation of ROS. The effect was evident after just 30 min of treatment.

Chelation of iron ions blocks the Fenton reaction and thus the production and the propagation of oxygen radicals - i.e. the species responsible for DNA and cellular damages - within the cells [31]. The presence of the iron chelator *o*-phenanthroline prevented the formation of DNA breaks and reduced DCFH oxidation (data not shown). Thus, as to nuclear DNA, the data described so far indicate that REH, according to previous data obtained in Jurkat cells [30], are prone to the DNA damaging action caused by SR, and that ROS are the species mediating this effect in both cell lines.

In the second part of our work, we treated REH cells with different concentration of SR in hypoxic conditions and analyzed the formation of ROS and the induction of apoptosis.

Fig. 6 shows the formation of ROS in normoxic and hypoxic treatment protocols. ROS formation was also observed in hypoxic conditions. The trend is similar in both conditions, although the formation of ROS is higher in normoxia (Fig. 6A). Of note, DNA damage was observed also in hypoxic conditions after treatment with SR (data not shown).

The proapoptotic effect of SR in hypoxia was lower than that observed in normoxia (Fig. 4). As an example, after treatment with SR 7.5 µM, we observed a four-fold increase with respect to controls in normoxia and a two-fold increase in hypoxia. At the highest concentration tested, we recorded a six-fold and about a three-fold increase with respect to controls in normoxia and hypoxia, respectively. After longer time of treatment (i.e. 48 h) in hypoxic conditions, SR mainly induced necrotic events. As an example, at SR 30 µM we recorded 30% of necrotic cells (vs. 10% in the control) and 20% of apoptotic cells (vs. 15% in the control). This trend was not observed in normoxic conditions, where at SR 30 µM the % of necrotic cells was 25% (vs. 8% in the control) and 44% of apoptotic cells (vs. 10% in the control).

**Figure 7:**
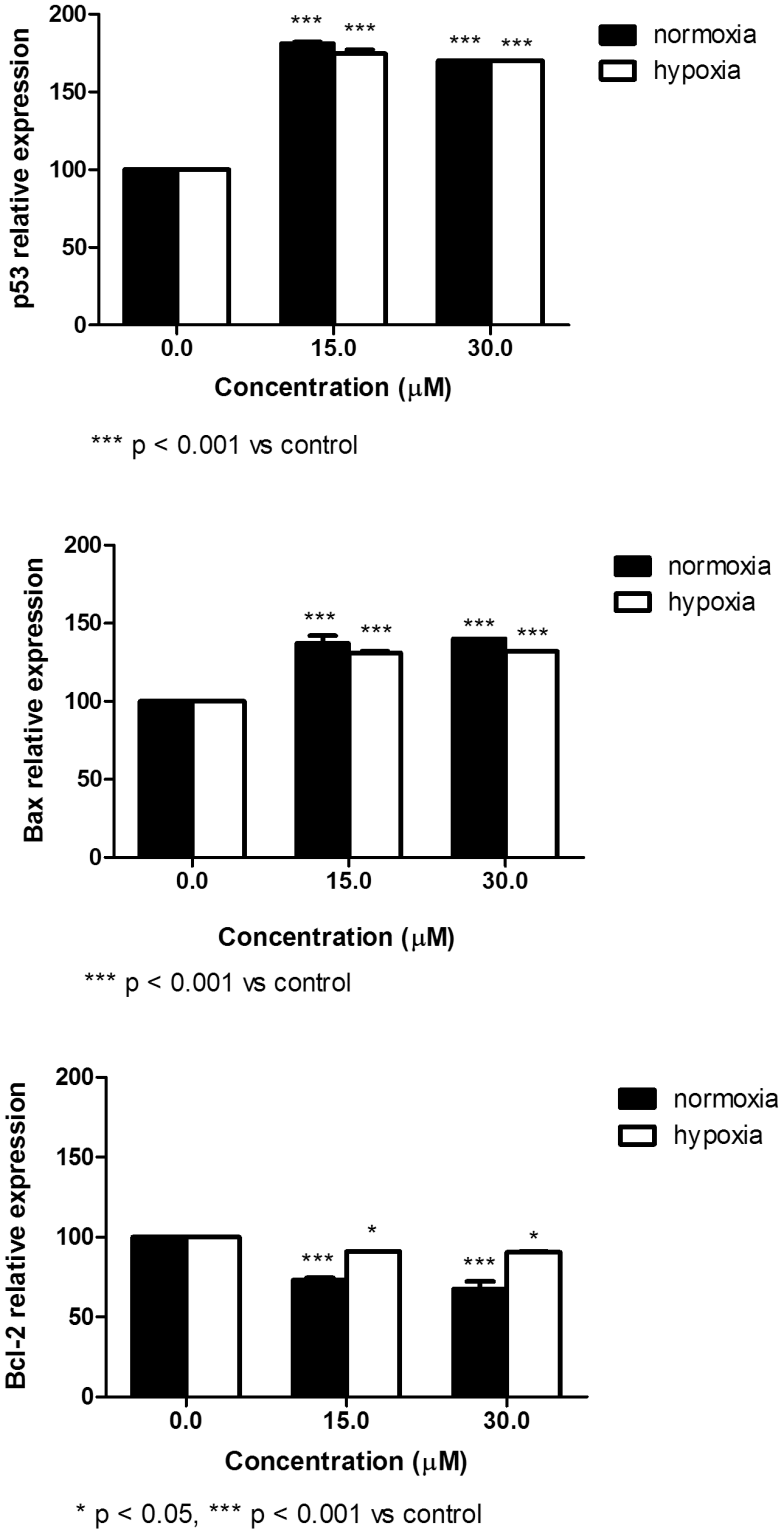
Effect of SR on p53, Bax and Bcl-2 protein expression determined in normoxic and hypoxic conditions on REH cells. Cells were treated with 0 to 30 µM SR for 24 h. Data are presented as mean ± SEM of at least four different experiments.

**Figure 8:**
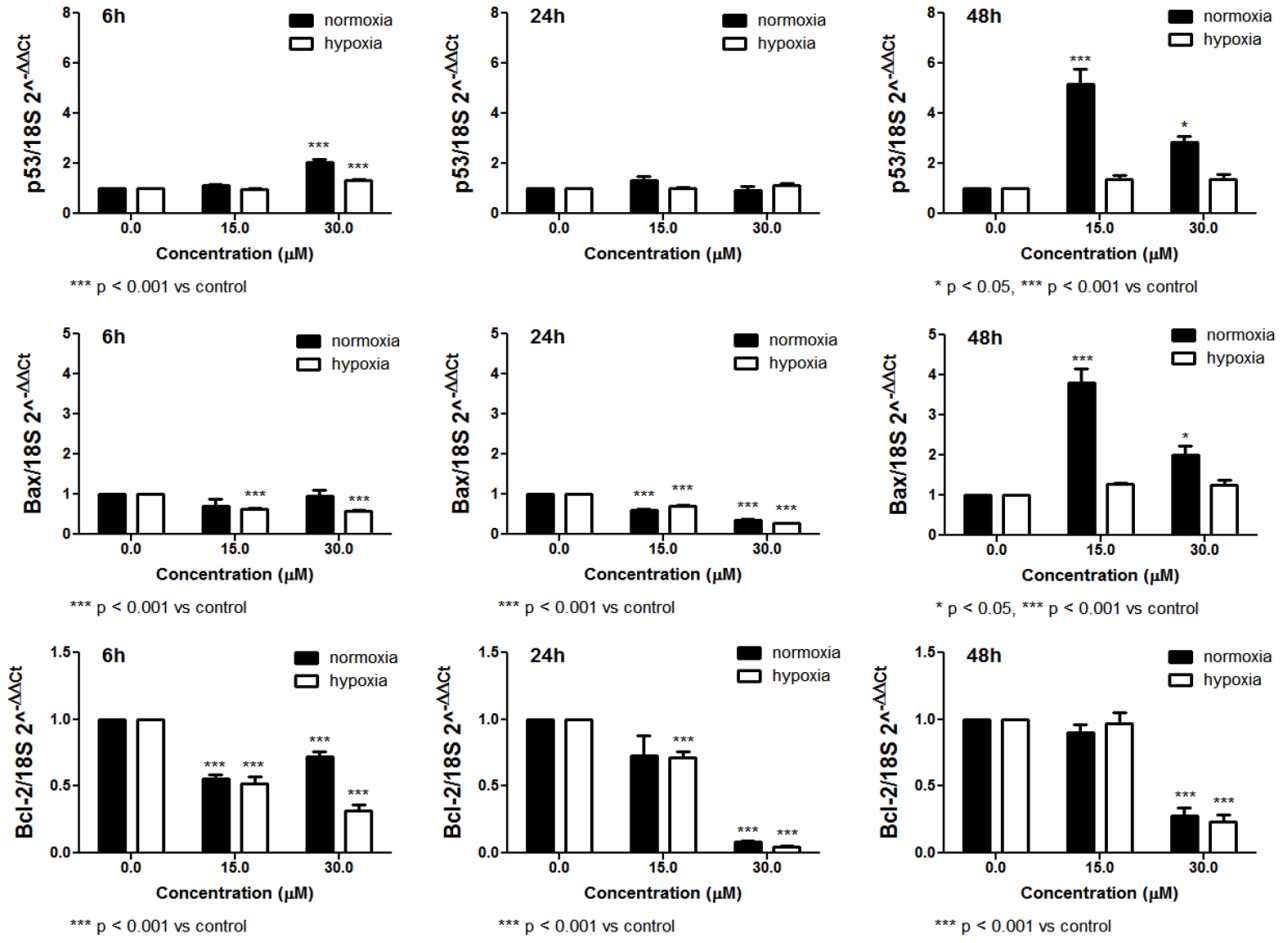
Effect of SR on p53, Bax and Bcl-2 mRNA relative expression calculated through the 2^-ΔΔC^t method and determined in normoxic and hypoxic conditions on REH cells. Cells were treated with 0 to 30 µM SR for 6, 24 or 48 h. Data are presented as mean ± SEM of at least four different experiments.

Survival of cancer cells is influenced by the interactions between pro- and anti-apoptotic proteins. We then analyzed the expression at mRNA and protein levels of some genes involved in the regulation of apoptosis. Following 48 h-treatment in normoxic conditions, SR induced a significant up-regulation of p53 and Bax protein expression (Fig. 7). However, the expression of the same genes at mRNA level seems more complex. After treatment with SR for 6, 24 or 48 h, we mainly observed an up-regulation of p53 expression (Fig. 8), while a down-regulation of Bax expression was recorded after 6 and 24 h of treatment. After 48 h, SR up-regulated Bax expression (Fig. 8). The expression of Bcl-2 was down-regulated at both mRNA and protein expression level and following 6, 24 or 48 h of exposure to SR (Figs. 7 and 8).

In hypoxic conditions, we observed a similar trend. However, the effects on p53 and Bax mRNA expression and the ratio of Bax/Bcl-2 proteins were less marked than in normoxia (Figs. 7 and 8).

**Figure 9:**
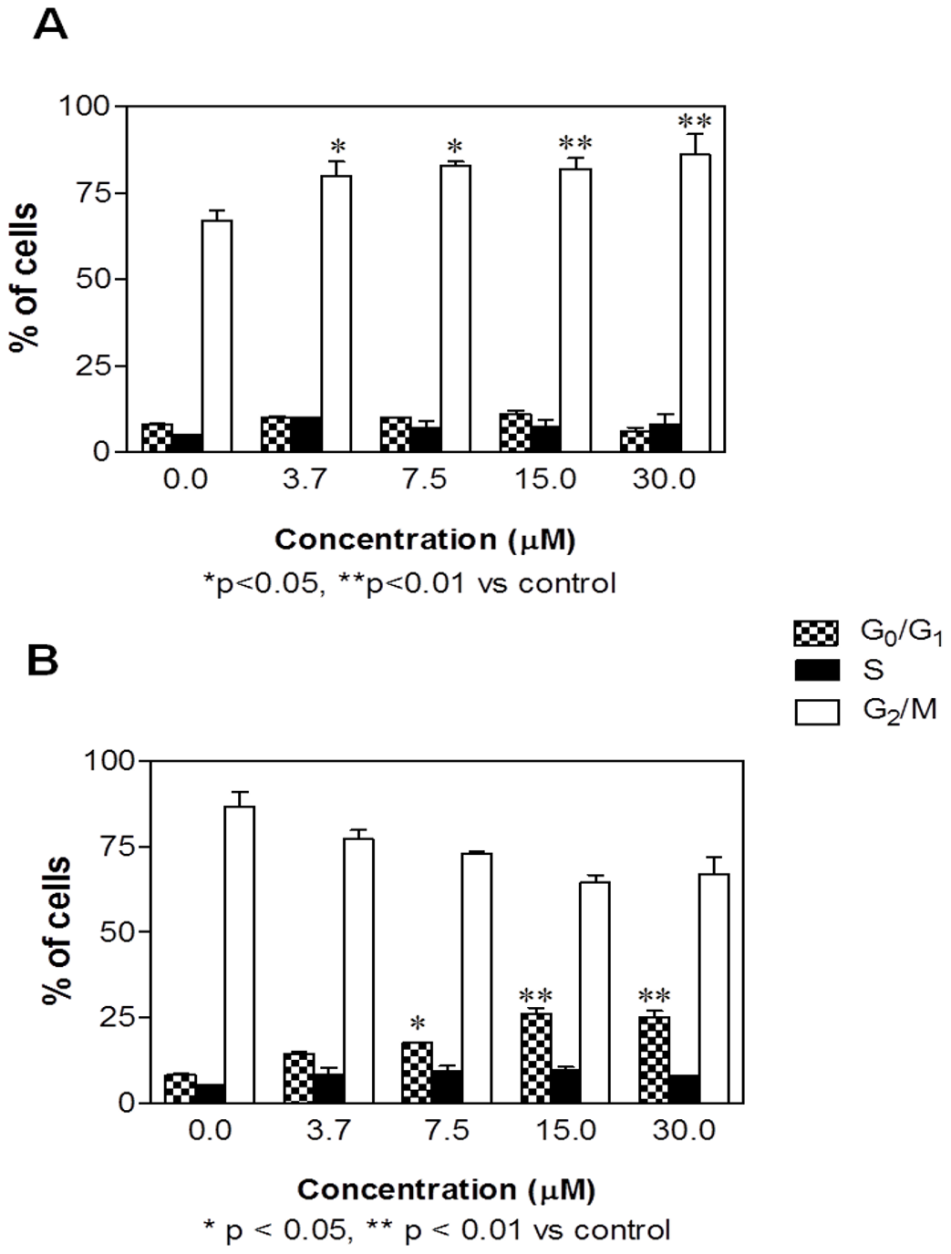
Cell-cycle distribution following 24 h culture in the absence or presence of SR in normoxic (A) and hypoxic (B) conditions on REH cells. Data are means ± SEM of four independent experiments.

The antileukemic effect of SR is strengthened by the inhibition of cell proliferation (Fig. 9) in both normoxic and hypoxic exposure to SR. In both conditions, the effect was recordable starting from the lowest concentration tested. However, the mechanism by which SR inhibits cell-cycle progression is different in the two treatment protocols. In normoxia, a block in G_2_/M phase was recorded, while an increase in G_1_ phase was observed in hypoxia.

## Discussion

The main goal of this study was to test the antileukemic properties of SR in hematological cancers. We found that synthesized SR caused a dose-dependent induction of apoptosis in acute leukemia cell lines and primary lymphoblasts from patients diagnosed with B-ALL, T-ALL, and AML.

Due to the possibility to easily obtain blast samples from leukemic patients, SR was tested on an *ex vivo* leukemia model. *Ex vivo* samples represent a quite good surrogate for determining the cellular response of the patient to the treatment and predicting the clinical outcome [32]–[34]. This can not be realized by using cell lines, which markedly differ from blasts directly taken from leukemic patients in terms of growth kinetics and pharmacological determinants [35]. By the *ex vivo* model and in accordance with previous data [20], [30], we confirmed that SR controlled the expansion of leukemic cells. However, our results show that SR can not be defined generically an antileukemic, as apparently it acts only on patients with particular types of leukemia. Indeed, we did not record any activity of SR on samples from CLL patients. CLL is generally described as a disease of failed apoptosis. Apoptosis resistance may stem from a combination of microenvironmental survival signals as well as from intrinsic alterations in the apoptotic machinery and deregulation of components of the DNA-damage response and repair pathways within the CLL cell [36]. Moreover, CD40 signaling has been recognized as a strong antiapoptotic pathway mediating drug resistance *in vitro*. All CLL cells express CD40, and *in vitro* resistance can be induced in 100% of patients [37]. The strong dependence on cellular and cytokine components of the microenvironment makes the *ex vivo* manipulation of CLL cells complex and resulting in biased findings [38]. Taking into account the absence of a cellular model for different types of CLL [39], further research might explore the effects of SR on blasts from CLL patients through the partial re-creation *in vitro* of the CLL microenvironment, including in particular leukemic cell survival signals.

One of the greatest problems for anticancer chemotherapy is chemoresistance, which is less frequent *ab initio* and has a major clinical impact during relapse. SR was tested also on non-responder patients. Although the number of patients was limited, it is interesting to note that SR retains its activity on this type of patients.

One of the more significant findings to emerge from this study is that SR exerts proapoptotic and antiproliferative effects in hypoxic conditions. Reduced oxygen tension affects cellular metabolism and the microenvironment, including pH level. These and other modulations caused by hypoxia can affect the response to pharmacological treatment [40]. Therefore, with the aim of gaining further mechanistic insight into the antileukemic activities of SR as well as the effects of the local environment on the outcome of treatment, we investigated the effects of SR in hypoxic conditions, which better mimics the tumor microenvironment *in vivo*. Our findings demonstrate that, despite hypoxic culture conditions, the antileukemic activity of SR is relatively preserved. We indeed recorded induction of apoptosis and production of ROS after treatment of leukemia cells with SR in both normoxic and hypoxic conditions. As to the mechanism through which SR induces apoptosis, an important trigger is likely to reside in its ability of damaging nuclear DNA. Indeed DNA damage is known as an event causally linked to the apoptotic commitment of target cells. The mechanism whereby DNA breaks are produced clearly involves the intracellular generation of ROS. Paradoxically, exposure of cells to low oxygen (hypoxia) leads to an increase in mitochondrial production of ROS, that are central upstream regulators of many of the cellular responses to hypoxia. Low levels of ROS are required for cellular processes such as proliferation and differentiation, while additional amounts of ROS above a certain threshold may cause cell-cycle arrest and/or apoptosis. The equilibrium of ROS concentration will thus be reached at the point where maximal signaling is permitted without causing irreversible damage to cellular components. Indeed, irreversible damage to cellular proteins, DNA, or lipids seems to be the case as compounds that raise ROS levels can effectively kill a variety of tumor cell lines [41]. Increasing ROS levels may indeed represent a therapeutic strategy to increase killing of cancer cells. Accordingly with those observations, in our experimental setting SR creates an oxidative cellular environment that induces DNA damage and Bax and p53 gene activation, which in turn helps trigger apoptosis. Indeed, the % of apoptotic cells and the up-regulation of p53 and Bax are particularly marked in normoxic conditions, where the production of ROS is higher than in hypoxia. However, the effect of SR on the mRNA expression of p53 and Bax is different, particularly for Bax, for which a down-regulation was observed after short times of treatment with SR. The quantification of both of these expression levels is not an exercise in redundancy. Indeed, analyses of mRNA and protein levels are complementary and both are necessary for a complete understanding of how the cell works. The different modulation of a gene at mRNA and protein levels caused by a xenobiotic can be due to different reasons, such as the poor definition of the complicated and varied post-transcriptional mechanisms involved in turning mRNA into protein, or the different half lives of proteins [42]. The differential effect of SR at mRNA and protein level seems not be imputable to an inhibition of proteasome. Instead, SR appears to stimulate proteasome [43], [44]. Alternately, a further study could assess the modulation by SR of microRNAs that moderate the p53 and Bax transcriptional program.

The results of this investigation show that SR is also able to inhibit cell-cycle progression through a block in the G_2_/M phase in normoxia and in the G_1_ phase in hypoxia. The G_2_/M block induced by SR in normoxia was reported in many cell lines [1]. The differential effect in hypoxic conditions is not surprising. As a matter of fact, cells subject to severe hypoxia will arrest in G_1_ or early S phase and cells in late S, G_2_ or M phase will finish cell division and arrest in G_1_, unless the hypoxia is severe [45].

As to the concentrations of SR used in this study, they could be not achievable from food supply. However, after a single oral dose of SR at 150 µmol/kg in rat, plasma concentrations of SR equivalents increase to 15.2 µM [46]. Furthermore, the potential development of SR in clinical practice could lead to the application of a drug delivery system to improve drug absorption.

In conclusion, *in vitro* and *ex vivo* experiments performed in the present study suggest that SR could represent an interesting therapeutic approach for patients affected by acute proliferative disorders and might set the stage for a novel therapeutic principle complementing our growing armature against malignancies. Large clinical trials will be necessary to confirm this attractive perspective *in vivo*.
